# Seroprevalence of SARS-CoV-2 in Guilan Province, Iran, April 2020

**DOI:** 10.3201/eid2702.201960

**Published:** 2021-02

**Authors:** Maryam Shakiba, Maryam Nazemipour, Arsalan Salari, Fardin Mehrabian, Seyed Saeed H. Nazari, Seyed Mahmoud Rezvani, Zahra Ghasempour, Abtin Heidarzadeh, Mohammad Ali Mansournia

**Affiliations:** Guilan University of Medical Sciences, Rasht, Iran (M. Shakiba, A. Salari, F. Mehrabian, S.M. Rezvani, Z. Ghasempour, A. Heidarzadeh);; Iran University of Medical Sciences, Tehran, Iran (M. Nazemipour);; Shahid Beheshti University of Medical Sciences, Tehran (S.S. Hashemi Nazari);; Tehran University of Medical Sciences, Tehran (M.A. Mansournia)

**Keywords:** asymptomatic infections, COVID-19, seroprevalence, coronavirus disease, SARS-CoV-2, severe acute respiratory syndrome coronavirus 2, viruses, respiratory infections, zoonoses, seroprevalence, Guilan, Iran

## Abstract

We determined the seroprevalence of severe acute respiratory syndrome coronavirus 2 (SARS-CoV-2) in an affected area in northern Iran in April 2020. Antibodies to SARS-CoV-2 were detected in 528 persons by using rapid tests. Adjusted prevalence of SARS-CoV-2 seropositivity was 22.2% (95% CI 16.4%–28.5%).

Coronavirus disease (COVID-19) was first reported in China and has now spread throughout the world. Global estimates of disease spread are based on confirmed cases in symptomatic patients (*1*). However, these estimates do not accurately reflect actual infection rates in the community because they exclude persons with mild or no symptoms or for whom testing is unavailable. Knowledge about actual infection rates is vital for accurately estimating the case-fatality rate, a public health measure of COVID-19 (*2*), and for projecting the course of the pandemic and determining public policy guidelines (*3*). 

Guilan Province was the second-largest province in Iran to have multiple confirmed cases of COVID-19 soon after the beginning of the pandemic. The epidemic curve has subsided in this province, making it an appropriate location to test for the presence of past infections through a seroprevalence survey. In this study, we provided a population-based seropositivity estimate of severe acute respiratory syndrome coronavirus 2 (SARS-CoV-2) infection based on World Health Organization protocol.

We conducted a cross-sectional population-based study among persons in Guilan Province during April 11–19, 2020. The study was approved by the Institutional Review Board of Guilan University of Medical Sciences (Rasht, Iran). All persons living in a household, regardless of age, were invited through multistage cluster random sampling. We selected clusters from the list of Comprehensive Healthcare Centers (CHCs) (the top units of the healthcare network in Iran) and used simple random sampling method to select households from those covered by CHCs. On the day participants arrived at the CHC, we took 10 µL capillary blood samples from each participant and collected information on demographics, disease history, COVID-19 symptoms in previous 3 months, and history of SARS-CoV-2 exposure. Samples were tested by using VivaDiag Rapid test kit (VivaChek, https://www.vivachek.com) for a SARS-CoV-2–specific serologic assay.

The design-adjusted prevalence of seropositivity was estimated by using inverse probability weighting with weights equal to the inverse of probability of selection for each participant (*4*). The prevalence estimates were then adjusted for test characteristics. We used a Monte Carlo bias analysis with 100,000 samples for sensitivity of 83.3% and specificity of 99% for IgM or IgG (*5*,*6*). The number of infections was calculated by multiplying infection prevalence by total population of Guilan Province. All analyses were performed in Stata version 14 (Stata, https://www.stata.com). Additional information about methods and results has been provided in the Appendix.

Of 632 households contacted, 196 households, consisting of a total of 551 persons, participated in this study. Eleven of those 551 participants refused blood sampling and could not be tested, and 12 had invalid test results. Of the remaining 528 participants, 117 were positive for either IgM or IgG (22.1% [95% CI 0.19%–0.26%]). Adjusted for design and test performance, prevalence was 22.2% (95% CI 16.4%–28.5%).

Seropositivity prevalence estimates varied most substantially according to age group, occupation, presence of COVID-19 symptoms in the previous 3 months, and county of residence (Table). Office workers had the highest prevalence of SARS-CoV-2 infection, followed by taxi drivers. Among counties, the highest prevalence of seropositivity was in Anzali, followed by Rasht. 

**Table:** Severe acute respiratory syndrome coronavirus 2 seropositivity prevalence estimates according to study variables, Guilan Province, Iran, April 2020*

Characteristic	Sample size (%), N = 528	No. positive	Design-adjusted prevalence (95% CI)	Design- and test performance–adjusted prevalence (95% CI†)
Sex				
M	257 (48.7)	55	16.8 (13.2–21.2)	19.0 (12.7–25.4)
F	271 (51.3)	62	22.2 (14.7–32.1)	25.6 (15.4–36.8)
Age group, y				
<5	26 (4.9)	4	8.7 (2.1–30.2)	9.8 (0.9–22.6)
5–17	101 (19.1)	20	17.0 (11.6–24.2)	19.1 (11.2–27.5)
18–59	329 (62.3)	74	21.0 (16.9–25.8)	24.1 (17.5–31.6)
≥60	72 (13.6)	19	22.4 (15.7–31.0)	25.7 (16.6–36.1)
Obesity, BMI >30				
No	474 (89.8)	107	19.8 (16.9–22.9)	22.6 (16.8–29.0)
Yes	54 (10.2)	10	15.4 (7.8–28.2)	17.3 (6.2–29.0)
SARS-CoV-2 exposure history				
No	452 (85.6)	95	18.1 (12.7–25.1)	20.4 (12.6–28.8)
Yes	76 (14.4)	22	26.9 (13.5–46.5)	31.2 (13.4–50.8)
COVID-19 symptoms in previous 3 mo				
No	382 (69.3)	65	15.3 (11.03–20.9)	17.2 (10.3–24.1)
Yes	169 (30.7)	52	30.05 (25.3–36.4)	35.5 (27.8–45.8)
Underlying condition				
No	420 (79.5)	89	18.2(13.6–24.03)	20.7 (13.5–28.3)
Yes	108 (20.5)	28	25.3 (18.3–33.9)	29.2 (19.8–40.2)
Place of residence				
Village	162 (30.7)	38	21.0 (16.0–27.1)	24.0 (16.5–32.4)
Town	366 (69.3)	79	19.2 (16.0–23.0)	21.9 (15.8–28.4)
Occupation‡				
Employee	53 (10.04)	19	46.0 (35.9–56.5)	54.3 (41.8–71.1)
Housekeeper	159 (30.1)	39	21.8 (13.4–33.5)	25.0 (13.6–37.5)
Student	114 (21.6)	22	15.6 (12.1–20.0)	17.5 (11.3–23.7)
Unemployed	67 (12.7)	11	11.8 (7.6–18.0)	12.9 (5.9–19.6)
Farmer	16 (3.03)	3	17.4 (9.9–28.8)	19.7 (9.1–31.0)
Salesman	46 (8.7)	5	7.9 (2.0–26.7)	8.7 (0.8–20.0)
Healthcare personnel	43 (8.1)	12	13.2 (6.5–24.9)	14.5 (4.5–25.0)
Taxi driver	13 (2.5)	5	24.0 (7.1–56.7)	28.0 (4.5–56.3)
Worker	17 (3.2)	1	2.5 (0.1–32.1)	28.0 (4.5–56.3)
County				
Rasht	226 (42.8)	56	20.8 (19.7–21.9)	23.7 (18.6–29.6)
Anzali	75 (14.2)	23	30.0 (29.7–30.4)	34.8 (29.7–43.2)
Astara	78 (14.8)	12	15.4 (14.3–16.6)	17.4 (12.0–21.8)
Lahijan	74 (14)	12	15.0 (13.6–16.5)	16.9 (11.5–21.4)
Rudbar	75 (14.2)	14	17.7 (15.5–20.2)	20.1 (14.5–25.7)

*BMI, body mass index; COVID-19, coronavirus disease 2019; SARS-CoV-2, severe acute respiratory syndrome coronavirus 2.  †Calculated using Monte Carlo simulation method.  ‡Employee was defined as a government employee working in an office. Worker was defined as a person performing manual jobs in nongovernmental locations.

In this study, the seroprevalence estimate of SARS-CoV-2 antibodies after adjusting for population and test characteristics was 22.2%. This result is much higher than those for previous seroprevalence estimates using an immunoassay test to detect antibodies in Spain (*7*); California, USA (*8*); and Geneva, Switzerland (*9*). Unlike Guilan Province, those places enacted severe lockdown policies to contain the pandemic, which might explain the higher prevalence of infection in our study. 

Our study’s limitations include possible selection bias if persons with previous COVID-19–like symptoms sought to participate in the study. However, in our study only 11 participants had a history of COVID-19 diagnosis. Otherwise, bias toward persons in good health who could participate in the study might result in an underestimation of actual prevalence. In addition, household sampling might result in an overestimation of prevalence compared with random sampling of persons because of clustering of infection in household contacts. We excluded persons in institutional residences (i.e., nursing homes, boarding schools, and prisons), for whom close contact with others might increase risk for infection, resulting in an underestimation of actual prevalence. Finally, our study used rapid test kits that have lower sensitivity than the ELISA test method, particularly for patients in the acute phase of infection. However, the study was designed to detect previous infection in healthy persons, in whom the test has better sensitivity. 

In conclusion, our findings imply that ≈518,000 persons in Guilan Province may have been infected with SARS-COV-2 as of April 19, 2020, which is substantially higher than the 1,600 cumulative confirmed cases recorded. As of May 3, if we assume a 3-week lag from time of infection to death (*10*), 625 persons had died of confirmed COVID-19 in Guilan Province. This number would correspond to an infection-fatality rate of 0.12%.

Appendix Additional information about seroprevalence of SARS-CoV-2 in Guilan Province, Iran, April 2020.
