# Supplementary material for: Seroprevalence of SARS-CoV-2 in Guilan Province, Iran, April 2020
Source: Emerg Infect Dis. 2021 Feb;27(2):636–8. doi: 10.3201/eid2702.201960 (PMC7853569; doi:10.3201/eid2702.201960)
Supplement: Appendix — Additional information about seroprevalence of SARS-CoV-2 in Guilan Province, Iran, April 2020. [file 20-1960-Techapp-s1.pdf]

Article DOI: https://doi.org/10.3201/eid2702.201960

Seroprevalence of SARS-CoV-2 in Guilan Province, Iran, April 2020

Appendix

Sampling

The sampling was conducted in the health service structure of Iran that had been exclusively organized during the COVID-19 pandemic as part of the National Mobilization Plan (NMP) against COVID-19. In Iran, public health services are provided through a nationwide referral network system, which following Health Transformation Plan had acceptable coverage in all urban and rural populations (*1*). This network started at rural and urban Comprehensive Health Care Centers (CHCs) in the periphery providing primary healthcare to a population residing in a predefined geographic area under the coverage of a CHC (*2*,*3*). In the NMP against COVID-19, a prevention strategy launched by the Ministry of Health for screening persons with COVID-19 symptoms (*4*), all households under coverage of CHCs were registered. Those who were not under coverage of CHCs were informed to register at the website salamat.gov.ir through extensive media advertisements. According to the report by Guilan University of Medical Sciences, this plan resulted in more than 92% coverage of households for screening of persons with COVID-19 symptoms (*5*). In this population-based cross-sectional study, a stratified multistage cluster random sampling approach was used to select participants. The strata were defined according to World Health Organization (WHO) protocol (*6*) for high- and low-incidence counties based on the ratio of hospitalized cases to total population in each county. The study was conducted in 3 high-incidence (Rasht, Anzali, Lahijan) and 2 low-incidence counties (Astara, Roudbar) as the primary sampling unit. Counties were randomly selected from 16 counties using simple random sampling method. At the second stage of sampling, clusters were selected from the list of urban and rural CHCs, and at the third stage of sampling, households were selected from the list of households under coverage of CHCs using simple random sampling method through computer generated random numbers. Households were defined as $\geq$2

persons living in the same place. The number of clusters in each county was assigned proportionally to the number of CHCs. Therefore, 15 CHCs were considered for Rasht county (the capital city of the province), and 5 CHCs were considered for each of the remaining 4 counties. Considering the almost equal population under coverage of CHCs, a sample size of 15 individuals/cluster was determined in the third stage of sampling by dividing total sample size by the total number of clusters.

Eligibility Criteria and Sample Size

Persons living in institutional residences, such as nursing homes, prisons, and boarding schools, and persons who refused to participate, were under active treatment for COVID-19, or who had contraindication to venipuncture were not invited to participate. With a prior prevalence of 50% for coronavirus infection based on closed cohort population (*7*), considering a 5% precision, design effect of 1.24 (*8*), and nonresponse rate of 10%, a total of 530 participants were considered for sample size. Guilan province is located in northern Iran and has a population of 2,354,848 over 16 counties.

Data Collection

Upon phones call to heads of the household (identified in households' electronic health records), a brief explanation of the research objectives was given and household members were asked to come to the CHC. For acknowledgment and to increase rates of participation, a package of incentives including ethanol alcohol, face masks, and kids' stickers were provided to household members. Appointments were scheduled at intervals to allow social distancing among households. After providing information about the risk and benefits of participation in the study, informed consent was acquired from head of household. An interviewer completed an electronic questionnaire collecting participants' demographic and exposure-history information. Sample collectors in personal protective equipment drew 10 µL of capillary blood into an EDTA–coated microtainer, and tubes were labeled with participant IDs. VivaDiag COVID-19 IgM/IgG from VivaChek (VivaChek, https://www.vivachek.com) was used for COVID-19 –specific serologic assay. According to manufacturer's instruction, 10 µL of serum or whole blood sample was added into the sample port followed by adding 2 to 3 drops (70–100 µL) of dilution buffer. Test

kits were read after ≈15 minutes. The sensitivity was 80% for IgG and both IgM and IgG, and 83% for IgM and either IgM or IgG (*9*) and the specificity was 100% (95% CI 95.7–100) For "IgM" and "IgM and IgG" and 99% (95% CI 94.2–100) for "IgM or IgG" and "IgG" (*10*).

Statistical Analysis

Demographic characteristics were described as frequency and percentage. The design-adjusted prevalence of COVID-19 positive test was estimated with consideration for clustering and unequal probability of sampling (*11*). Since the probability of selection varied over the participants, inverse probability weighting is required to adjust for selection bias. The weight formula was $\frac{1}{P_1 \times P_2 \times P_3}$, where $P_1 \times P_2 \times P_3$ are probability of selection for each participant, which is equal to the multiplication of the probabilities of selection at each of the 3 stages of sampling (i.e., county, CHC, and household). The highest level of clustering or primary sampling unit (county) was considered in the calculation of CI using Taylor-linearized standard error. The prevalence estimates were further adjusted for Rapid test sensitivity (Se) and specificity (Sp) using the following formula (*12,13*):

TP = (AP + Sp - 1) / (Se + Sp - 1)

where TP denotes true prevalence and AP denotes apparent prevalence. For test performance adjustment, we used the results of previously published papers (*9,10*). We used a Monte Carlo bias analysis with 100,000 samples for sensitivity (*14,15*): β distribution with parameters a = 25, b = 5 for seropositivity for "IgM or IgG" and "IgM" (Se = 83.3%), and another β distribution with parameters a = 24, b = 6 for "IgM and IgG" and "IgG" (Se = 80%). We performed a similar bias analysis for specificity of 100% (95% CI 95.7–100) for "IgM" and "IgM and IgG" and 99% (95% CI 94.2–100) for "IgM or IgG" and "IgG" using β distribution with parameters a = 47.84, b = 0.36 and a = 47.84, b = 0.64, respectively. For sensitivity, we set α = the number of true positives and β = the number of false negatives in the reference (*9*) in the Appendix so that the mean and variance of β distribution approximately equal the mean and variance of sensitivity estimates in reference (*9*). For specificity, we chose α and β values so that the percentiles 2.5 and 97.5 of β distribution exactly match the 95% confidence limits of specificity in reference (*10*) using a grid search. The apparent prevalence was drawn from a Normal distribution with mean and standard deviation equal to design-adjusted prevalence

estimate and its standard error, respectively. We derived the point estimates and 95% simulation intervals (for simplicity, called confidence intervals in this paper) using the median and 2.5th and 97.5th percentiles of Monte Carlo distribution. All analyses were performed in Stata version 14 (Stata, https://www.stata.com/).

Supplementary Results

In this study, the major reasons for nonparticipation were concerns about acquiring infection in the CHC (17%), busy schedule (20%), lack of assurance to system (2%), no response (31%), and other nonspecified reasons (28%). The distribution of nonparticipation was not substantially different among the counties and ranged between 30% to 33%. The variable distribution in the sample was not substantially different from that of the province except for place of residence (32% in village, 43% in province) (Appendix Table 1).

Supplementary Discussion

The current result is much higher than previous seroprevalence estimates in California (*16*), Spain (*17*), and Geneva (*18*), which were lower than 10%. Our estimate is closer to findings from France (A. Fontanet, unpub. data, https://doi.org/10.1101/2020.04.18.20071134) and Germany (H. Streeck, unpub. data, https://doi.org/10.1101/2020.05.04.20090076), which were ≈20%. Prevalence of infection across space and time varies greatly. Geneva repeated cross-sectional seroprevalence studies showing an increase from 4.8% in the first week to 10.8% in the fifth week (*18*). For one-time cross-sectional investigation, WHO recommended the survey be conducted after the peak of transmission of the epidemic wave (*6*). This study was conducted during April 11–19 after the peak of the epidemic wave in Guilan province, which had occurred in early April. Study design based on individual or household sample might also influence the results. In the household sampling that formed the basis of our study, we expect an overestimation of seroprevalence, assuming that 1 infection in a household would increase the likelihood of other infections more than 1 infection in random persons. Another factor that might explain the heterogeneity of the reported prevalence of infection among communities is the severity of lockdown policies enacted by different societies to contain the pandemic. In Guilan province, except for school and university closures, the restriction policy was not stringent and

could have resulted in higher rates of infection. The high seroprevalence of COVID-19 in Guilan province might also be related to an economic relationship with China in a free trade zone in northern Guilan province. The zone is in the county of Anzali, which had the highest seroprevalence in Guilan province.

In this study, prevalence of infection in children <5 years of age was 9.8%. For half of seropositive children, no previous COVID-19–associated symptoms were reported. This finding might support the hypothesis that more children might be infected than previously thought. In the current study, the overall prevalence in children and adolescents (0–19 years of age) was 14%. In 2 previous studies, a rate of 3.4% in Spain and 9.6% in Switzerland were reported for similar age groups (*17*,*18*). In contrast to a previous study in Geneva with a low seroprevalence among children 5–9 years of age and persons >65 years of age (*18*), we found that persons ≥60 years of age had the highest rate of infection. During the pandemic, schools and universities were closed and the elderly were asked to shelter in place. As to infection rates among children, more studies are needed to clarify the immunologic responses of children to COVID-19.

Employees and taxi drivers were the two occupations that had the highest prevalence of infection compared to other job categories. These jobs require many encounters with other persons. Employees work in governmental offices and interact with many clients during their jobs. Bank employees, also included in this group, are exposed to paper money (a source of infection during the pandemic) in addition to having contact with many clients. In Iran, taxi drivers drive up to 4 persons/trip, making them a high-risk group for COVID-19.

This study found an infection fatality rate of 0.12%, similar to previous seroprevalence studies that estimated rates of between 0.03%–0.5% (J. Ioannidis, unpub. data, https://doi.org/10.1101/2020.05.13.20101253). However, the estimated infection fatality rate is much lower than currently reported estimates of case fatality rate for COVID-19 of between 3%–4% (*19*). Previous estimate of case fatality rate using lag time for fatality in China was between 0.25%–3.0% (*20*).

References

1. Moradi-Lakeh M, Vosoogh-Moghaddam A. Health sector evolution plan in Iran; equity and sustainability concerns. Int J Health Policy Manag. 2015;4:637–40. PubMed https://doi.org/10.15171/ijhpm.2015.160

2. Sajadi HS, Majdzadeh R. From primary health care to universal health coverage in the Islamic Republic of Iran: a journey of four decades. Arch Iran Med. 2019;22:262–8. PubMed

3. Shadpour K. Primary health care networks in the Islamic Republic of Iran. East Mediterr Health J. 2000;6:822–5. PubMed

4. Raeisi A, Tabrizi JS, Gouya MM. IR of Iran National Mobilization against COVID-19 epidemic. Arch Iran Med. 2020;23:216–9. PubMed https://doi.org/10.34172/aim.2020.01

5. Salari A. Screening of more than 92% of households in Guilan province in National Mobilization Plan against COVID-19. 2020 [cited 2020 Aug 19]. https://www.gums.ac.ir/pharmacy/Page.aspx?mID=4777&Page=News/Shownews&NewsId=58229&NewsDate=13990119

6. World Health Organization. Population-based age-stratified seroepidemiological investigation protocol for COVID-19 virus infection 2020 [cited 2020 Apr 1]. https://www.who.int/publications-detail/population-based-age-stratified-seroepidemiological-investigation-protocol-for-covid-19-virus-infection

7. Oran DP, Topol EJ. Prevalence of asymptomatic SARS-CoV-2 infection: a narrative review. Ann Intern Med. 2020;173:362–7. PubMed https://doi.org/10.7326/M20-3012

8. Campbell MK, Fayers PM, Grimshaw JM. Determinants of the intracluster correlation coefficient in cluster randomized trials: the case of implementation research. Clin Trials. 2005;2:99–107. PubMed https://doi.org/10.1191/1740774505cn071oa

9. Cassaniti I, Novazzi F, Giardina F, Salinaro F, Sachs M, Perlini S, et al.; Members of the San Matteo Pavia COVID-19 Task Force. Performance of VivaDiag COVID-19 IgM/IgG Rapid Test is inadequate for diagnosis of COVID-19 in acute patients referring to emergency room department. J Med Virol. 2020;92:1724–7. PubMed https://doi.org/10.1002/jmv.25800

10. Van Elslande J, Houben E, Depypere M, Brackenier A, Desmet S, André E, et al. Diagnostic performance of seven rapid IgG/IgM antibody tests and the Euroimmun IgA/IgG ELISA in COVID-19 patients. Clin Microbiol Infect. 2020;26:1082–7. PubMed https://doi.org/10.1016/j.cmi.2020.05.023

11. Mansournia MA, Altman DG. Inverse probability weighting. BMJ. 2016;352:i189. PubMed https://doi.org/10.1136/bmj.i189

12. Greenland S, Lash T. Bias analysis. In: Rothman K, Greenland S, Lash T, editors. Modern epidemiology. 3 edition. Philadelphia: Lippincott Williams & Wilkins; 2008. p. 345–80.

13. Lash T, Fox M, Fink A. Applying quantitative bias analysis to epidemiologic data. New York: Springer; 2009.

14. Mirzazadeh A, Mansournia MA, Nedjat S, Navadeh S, McFarland W, Haghdoost AA, et al. Bias analysis to improve monitoring an HIV epidemic and its response: approach and application to a survey of female sex workers in Iran. J Epidemiol Community Health. 2013;67:882–7. PubMed https://doi.org/10.1136/jech-2013-202521

15. Pakzad R, Nedjat S, Yaseri M, Salehiniya H, Mansournia N, Nazemipour M, et al. Effect of smoking on breast cancer by adjusting for smoking misclassification bias and confounders using a probabilistic bias analysis method. Clin Epidemiol. 2020;12:557–68. PubMed https://doi.org/10.2147/CLEP.S252025

16. Sood N, Simon P, Ebner P, Eichner D, Reynolds J, Bendavid E, et al. Seroprevalence of SARS-CoV-2-specific antibodies among adults in Los Angeles County, California, on April 10–11, 2020. JAMA. 2020;323:2425–7. PubMed https://doi.org/10.1001/jama.2020.8279

17. Pollán M, Pérez-Gómez B, Pastor-Barriuso R, Oteo J, Hernán MA, Pérez-Olmeda M, et al.; ENE-COVID Study Group. Prevalence of SARS-CoV-2 in Spain (ENE-COVID): a nationwide, population-based seroepidemiological study. Lancet. 2020;396:535–44. PubMed https://doi.org/10.1016/S0140-6736(20)31483-5

18. Stringhini S, Wisniak A, Piumatti G, Azman AS, Lauer SA, Baysson H, et al. Seroprevalence of anti-SARS-CoV-2 IgG antibodies in Geneva, Switzerland (SEROCoV-POP): a population-based study. Lancet. 2020;396:313–9. PubMed https://doi.org/10.1016/S0140-6736(20)31304-0

19. World Health Organization. Coronavirus disease 2019 (COVID-19) situation report 2020 [cited 2020 Aug 14]. https://www.who.int/docs/default-source/coronaviruse/situation-reports/20200306-sitrep-46-covid-19.pdf?sfvrsn=96b04adf_4

20. Wilson N, Kvalsvig A, Barnard LT, Baker MG. Case-fatality risk estimates for COVID-19 calculated by using a lag time for fatality. Emerg Infect Dis. 2020;26:1339–441. PubMed https://doi.org/10.3201/eid2606.200320

Appendix Table 1. Demographic characteristics of sample relative to Guilan Province population

Characteristics	Sample no. (%)	Guilan Province (%)
Sex		
M	27(49)	50
F	281(51)	50
Age group		
<5	27(5)	6
5–17	107(19)	17
18–59	343(62)	63
≥60	74(13)	14
Living place		
Village	175(32)	43
City	376(68)	56

Appendix Table 2. Severe acute respiratory syndrome coronavirus 2 seropositivity prevalence estimates in Guilan province, April 2020

Antibody seropositivity	No. (%)	Design-adjusted prevalence (95% CI)	Design- and test performance-adjusted prevalence (95% CI*)
IgM	102 (19.3)	17.6% (13.4–22.7)	20.4% (14.3–27.4)
IgG	113 (21.4)	18.9% (15.8–22.4)	22.3% (16.1–29.5)
IgM and IgG	98 (18.6)	16.7% (12.2–22.3)	20.1% (13.3–28.0)
IgM or IgG	117 (22.1)	19.4% (16.5–22.7)	22.2% (16.4–28.5)

*CI calculated using Monte Carlo simulation method.
